# Bis[(*E*)-4-bromo-2-(ethoxy­imino­meth­yl)phenolato-κ^2^ *N*,*O* ^1^]copper(II)

**DOI:** 10.1107/S160053680904433X

**Published:** 2009-10-31

**Authors:** Shang-Sheng Gong, Wen-Kui Dong, Jun-Feng Tong, Li Li, Jian-Chao Wu

**Affiliations:** a School of Chemical and Biological Engineering, Lanzhou Jiaotong University, Lanzhou 730070, People’s Republic of China

## Abstract

The title compound, [Cu(C_9_H_9_BrNO_2_)_2_], is a centrosymmetric mononuclear copper(II) complex. The Cu atom is four-coordinated in a *trans*-CuN_2_O_2_ square-planar geometry by two phenolate O and two oxime N atoms from two symmetry-related *N*,*O*-bidentate (*E*)-4-bromo-2-(ethoxy­imino­meth­yl)phenolate oxime-type ligands. An inter­esting feature of the crystal structure is the centrosymmetric inter­molecular Cu⋯O inter­action [3.382 (1) Å], which establishes an infinite chain structure along the *b* axis.

## Related literature

For background to oximes, see: Cervera *et al.* (1997 ▶); Chaudhuri, (2003 ▶); Costes *et al.* (1998 ▶); Kukushkin *et al.* (1996 ▶). For related structures, see: Dong *et al.* (2009 ▶). For the synthesis, see: Wang *et al.* (2008 ▶); Zhao *et al.* (2009 ▶).
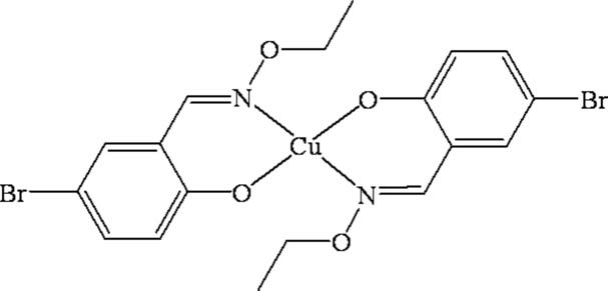


## Experimental

### 

#### Crystal data

[Cu(C_9_H_9_BrNO_2_)_2_]
*M* *_r_* = 549.70
Monoclinic, 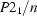

*a* = 10.0682 (13) Å
*b* = 5.4998 (8) Å
*c* = 17.990 (2) Å
β = 96.846 (1)°
*V* = 989.1 (2) Å^3^
*Z* = 2
Mo *K*α radiation
μ = 5.17 mm^−1^
*T* = 298 K
0.41 × 0.21 × 0.14 mm

#### Data collection

Bruker SMART 1000 diffractometer
Absorption correction: multi-scan (*SADABS*; Sheldrick, 1996 ▶) *T*_min_ = 0.226, *T*_max_ = 0.531
4684 measured reflections
1741 independent reflections
1356 reflections with *I* > 2σ(*I*)
*R*_int_ = 0.041

#### Refinement

*R*[*F* ^2^ > 2σ(*F* ^2^)] = 0.031
*wR*(*F* ^2^) = 0.080
*S* = 1.01
1741 reflections
125 parameters
H-atom parameters constrained
Δρ_max_ = 0.25 e Å^−3^
Δρ_min_ = −0.55 e Å^−3^

### 

Data collection: *SMART* (Siemens, 1996 ▶); cell refinement: *SAINT* (Siemens, 1996 ▶); data reduction: *SAINT*; program(s) used to solve structure: *SHELXS97* (Sheldrick, 2008 ▶); program(s) used to refine structure: *SHELXL97* (Sheldrick, 2008 ▶); molecular graphics: *SHELXTL* (Sheldrick, 2008 ▶); software used to prepare material for publication: *SHELXTL*.

## Supplementary Material

Crystal structure: contains datablocks global, I. DOI: 10.1107/S160053680904433X/om2289sup1.cif

Structure factors: contains datablocks I. DOI: 10.1107/S160053680904433X/om2289Isup2.hkl

Additional supplementary materials:  crystallographic information; 3D view; checkCIF report

## Figures and Tables

**Fig. 1.**
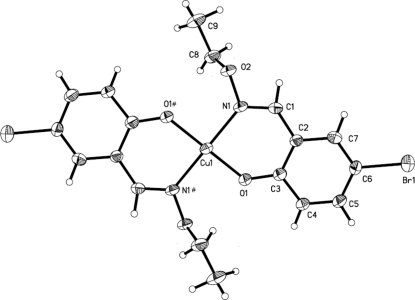
The molecular structure of the title compound with the atom numbering scheme [Symmetry codes: -x + 1,-y + 1,-z + 1]. Unlabelled atoms are related to their labelled counterparts by the inversion operation. Displacement ellipsoids for non-hydrogen atoms are drawn at the 30% probability level.

**Fig. 2.**
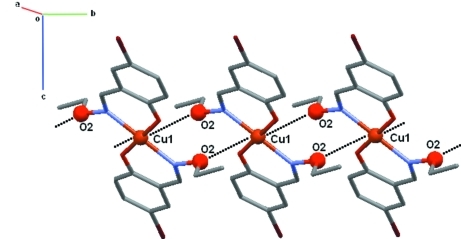
Packing diagram for the title compound, showing an infinite one-dimensional chain structure formed by short Cu···O contact viewed along the b axis. H atoms not involved in hydrogen bonding have been omitted for clarity.

## supplementary crystallographic information

### Comment

Oximes are a traditional class of chelating ligands widely used in coordination and analytical chemistry and extraction metallurgy (Kukushkin *et al.*, 1996; Chaudhuri, 2003). Due to their marked ability to from bridges between metal ions, oxime-containing ligands may be used to obtain polynuclear compounds in the field of molecular magnetism and supramolecular chemistry (Cervera *et al.*, 1997; Costes *et al.*, 1998). As a continuation of our study (Wang *et al.*, 2008; Zhao *et al.*, 2009) on oxime-type compounds, the title mononuclear copper(II) complex (Fig. 1), is reported in this paper.

The title compound is a centrosymmetric mononuclear copper(II) complex. The copper(II) ion, lying on the inversion centre, is four-coordinated in a *trans*-CuN_2_O_2_ square-planar geometry, with two phenolate O and two oxime N atoms from two N,*O*-bidentate oxime-type ligands. All bond lengths and angles are within normal ranges. The Cu—O and Cu—N bond lengths are 1.880 (2) Å and 1.994 (3) Å, respectively, which are comparable to those observed in a similar Schiff base copper(II) complex (Dong *et al.*, 2009).

The interesting feature of the crystal structure, as shown in Fig. 2, is the centrosymmetric intermolecular Cu···O [3.382 (1) Å] interaction, which forms an infinite one-dimensional chain structure along the *b* axis.

### Experimental

(*E*)-5-Bromo-2-hydroxybenzaldehyde *O*-ethyl oxime (HL) was synthesized according to the analogous method (Wang *et al.*, 2008; Zhao *et al.*, 2009). A blue solution of copper(II) acetate monohydrate (1.7 mg, 0.008 mmol) in methanol (3 ml) was added dropwise to a solution of HL (2.1 mg, 0.009 mmol) in methanol (4 ml) at room temperature. The color of the mixing solution turned to yellow immediately, then turned to brown slowly and was allowed to stand at room temperature for several days. With evaporation of the solvent, dark-brown needle-like single crystals suitable for X-ray crystallographic analysis were obtained. IR: ν C=N, 1608 cm^-1^, ν Ar—O, 1242 cm^-1^, ν Cu—N, 445 cm^-1^ and ν Cu—O, 424 cm^-1^. Yield, 47.1%. Anal. Calcd. for C_18_H_18_Br_2_CuN_2_O_4_: C, 39.33; H, 3.30; Cu, 11.56; N, 5.10. Found: C, 39.20; H, 3.38; Cu, 11.62; N, 4.87.

### Refinement

Non-H atoms were refined anisotropically. H atoms were treated as riding atoms with distances C—H = 0.96 Å (CH_3_), 0.97 Å (CH_2_) and 0.93 Å (CH). The isotropic displacement parameters for all H atoms were set equal to 1.2 or 1.5 *U*_eq_ of the carrier atom.

### Crystal data

**Table:**

[Cu(C_9_H_9_BrNO_2_)_2_]	*F*(000) = 542
*M**_r_* = 549.70	*D*_x_ = 1.846 Mg m^−^^3^
Monoclinic, *P*2_1_/*n*	Mo *K*α radiation, λ = 0.71073 Å
Hall symbol: -P 2yn	Cell parameters from 1900 reflections
*a* = 10.0682 (13) Å	θ = 2.2–25.1°
*b* = 5.4998 (8) Å	µ = 5.17 mm^−^^1^
*c* = 17.990 (2) Å	*T* = 298 K
β = 96.846 (1)°	Needle-shaped, black
*V* = 989.1 (2) Å^3^	0.41 × 0.21 × 0.14 mm
*Z* = 2	

### Data collection

**Table:**

Bruker SMART 1000 diffractometer	1741 independent reflections
Radiation source: fine-focus sealed tube	1356 reflections with *I* > 2σ(*I*)
graphite	*R*_int_ = 0.041
φ and ω scans	θ_max_ = 25.0°, θ_min_ = 2.2°
Absorption correction: multi-scan (*SADABS*; Sheldrick, 1996)	*h* = −11→7
*T*_min_ = 0.226, *T*_max_ = 0.531	*k* = −6→6
4684 measured reflections	*l* = −21→21

### Refinement

**Table:**

Refinement on *F*^2^	Primary atom site location: structure-invariant direct methods
Least-squares matrix: full	Secondary atom site location: difference Fourier map
*R*[*F*^2^ > 2σ(*F*^2^)] = 0.031	Hydrogen site location: inferred from neighbouring sites
*wR*(*F*^2^) = 0.080	H-atom parameters constrained
*S* = 1.01	*w* = 1/[σ^2^(*F*_o_^2^) + (0.0416*P*)^2^ + 0.0351*P*] where *P* = (*F*_o_^2^ + 2*F*_c_^2^)/3
1741 reflections	(Δ/σ)_max_ < 0.001
125 parameters	Δρ_max_ = 0.25 e Å^−^^3^
0 restraints	Δρ_min_ = −0.55 e Å^−^^3^

### Special details

**Table:**

Geometry. All e.s.d.'s (except the e.s.d. in the dihedral angle between two l.s. planes) are estimated using the full covariance matrix. The cell e.s.d.'s are taken into account individually in the estimation of e.s.d.'s in distances, angles and torsion angles; correlations between e.s.d.'s in cell parameters are only used when they are defined by crystal symmetry. An approximate (isotropic) treatment of cell e.s.d.'s is used for estimating e.s.d.'s involving l.s. planes.
Refinement. Refinement of *F*^2^ against ALL reflections. The weighted *R*-factor *wR* and goodness of fit *S* are based on *F*^2^, conventional *R*-factors *R* are based on *F*, with *F* set to zero for negative *F*^2^. The threshold expression of *F*^2^ > σ(*F*^2^) is used only for calculating *R*-factors(gt) *etc*. and is not relevant to the choice of reflections for refinement. *R*-factors based on *F*^2^ are statistically about twice as large as those based on *F*, and *R*- factors based on ALL data will be even larger.

### Fractional atomic coordinates and isotropic or equivalent isotropic displacement parameters (Å^2^)

**Table:**

	*x*	*y*	*z*	*U*_iso_*/*U*_eq_	
Cu1	0.5000	0.5000	0.5000	0.04220 (19)	
Br1	−0.06756 (4)	0.09316 (8)	0.22617 (2)	0.06250 (18)	
N1	0.5039 (2)	0.2157 (5)	0.43148 (14)	0.0399 (6)	
O1	0.3323 (2)	0.5904 (4)	0.45038 (14)	0.0573 (7)	
O2	0.6088 (2)	0.0414 (4)	0.44170 (12)	0.0463 (6)	
C1	0.4064 (3)	0.1341 (6)	0.38559 (17)	0.0408 (8)	
H1	0.4186	−0.0143	0.3625	0.049*	
C2	0.2803 (3)	0.2546 (6)	0.36725 (16)	0.0374 (7)	
C3	0.2497 (3)	0.4733 (6)	0.40143 (19)	0.0441 (8)	
C4	0.1206 (3)	0.5724 (7)	0.3809 (2)	0.0550 (10)	
H4	0.0977	0.7171	0.4029	0.066*	
C5	0.0289 (3)	0.4618 (7)	0.3298 (2)	0.0508 (9)	
H5	−0.0552	0.5307	0.3176	0.061*	
C6	0.0613 (3)	0.2477 (6)	0.29645 (18)	0.0420 (8)	
C7	0.1851 (3)	0.1453 (6)	0.31422 (18)	0.0439 (8)	
H7	0.2062	0.0018	0.2909	0.053*	
C8	0.7242 (3)	0.1298 (7)	0.4114 (2)	0.0545 (10)	
H8A	0.7488	0.2894	0.4315	0.065*	
H8B	0.7070	0.1416	0.3573	0.065*	
C9	0.8336 (4)	−0.0500 (8)	0.4337 (2)	0.0677 (12)	
H9A	0.8477	−0.0626	0.4873	0.102*	
H9B	0.9146	0.0034	0.4156	0.102*	
H9C	0.8085	−0.2061	0.4126	0.102*	

### Atomic displacement parameters (Å^2^)

**Table:**

	*U*^11^	*U*^22^	*U*^33^	*U*^12^	*U*^13^	*U*^23^
Cu1	0.0333 (3)	0.0474 (4)	0.0453 (4)	0.0111 (3)	0.0021 (2)	−0.0040 (3)
Br1	0.0549 (3)	0.0701 (3)	0.0589 (3)	−0.00385 (19)	−0.00800 (18)	−0.00911 (19)
N1	0.0340 (14)	0.0437 (17)	0.0429 (16)	0.0140 (12)	0.0079 (12)	0.0043 (13)
O1	0.0416 (13)	0.0519 (16)	0.0738 (17)	0.0155 (11)	−0.0115 (12)	−0.0206 (13)
O2	0.0385 (12)	0.0467 (14)	0.0542 (14)	0.0163 (11)	0.0083 (10)	0.0033 (11)
C1	0.0415 (18)	0.040 (2)	0.0425 (19)	0.0084 (15)	0.0117 (15)	−0.0025 (15)
C2	0.0345 (16)	0.042 (2)	0.0368 (18)	0.0042 (14)	0.0067 (13)	0.0007 (14)
C3	0.0372 (18)	0.045 (2)	0.050 (2)	0.0038 (16)	0.0046 (15)	−0.0013 (17)
C4	0.0411 (19)	0.050 (2)	0.070 (3)	0.0150 (17)	−0.0076 (17)	−0.0134 (19)
C5	0.0367 (18)	0.050 (2)	0.064 (2)	0.0097 (16)	−0.0010 (16)	−0.0011 (19)
C6	0.0356 (17)	0.051 (2)	0.0390 (18)	−0.0034 (15)	0.0003 (14)	0.0010 (16)
C7	0.049 (2)	0.043 (2)	0.0414 (19)	0.0039 (16)	0.0126 (16)	−0.0029 (16)
C8	0.0437 (19)	0.069 (3)	0.053 (2)	0.0164 (18)	0.0150 (17)	0.0045 (19)
C9	0.045 (2)	0.081 (3)	0.079 (3)	0.025 (2)	0.016 (2)	0.013 (2)

### Geometric parameters (Å, °)

**Table:**

Cu1—O1^i^	1.880 (2)	C3—C4	1.417 (4)
Cu1—O1	1.880 (2)	C4—C5	1.366 (5)
Cu1—N1^i^	1.994 (3)	C4—H4	0.9300
Cu1—N1	1.994 (3)	C5—C6	1.378 (5)
Br1—C6	1.901 (3)	C5—H5	0.9300
N1—C1	1.285 (4)	C6—C7	1.370 (4)
N1—O2	1.422 (3)	C7—H7	0.9300
O1—C3	1.307 (4)	C8—C9	1.499 (5)
O2—C8	1.427 (4)	C8—H8A	0.9700
C1—C2	1.435 (4)	C8—H8B	0.9700
C1—H1	0.9300	C9—H9A	0.9600
C2—C3	1.402 (4)	C9—H9B	0.9600
C2—C7	1.405 (4)	C9—H9C	0.9600
			
O1^i^—Cu1—O1	180.000 (1)	C3—C4—H4	119.0
O1^i^—Cu1—N1^i^	89.80 (10)	C4—C5—C6	119.8 (3)
O1—Cu1—N1^i^	90.20 (10)	C4—C5—H5	120.1
O1^i^—Cu1—N1	90.20 (10)	C6—C5—H5	120.1
O1—Cu1—N1	89.80 (10)	C7—C6—C5	120.4 (3)
N1^i^—Cu1—N1	180.0	C7—C6—Br1	120.0 (3)
C1—N1—O2	110.2 (2)	C5—C6—Br1	119.6 (2)
C1—N1—Cu1	127.0 (2)	C6—C7—C2	120.7 (3)
O2—N1—Cu1	121.18 (18)	C6—C7—H7	119.6
C3—O1—Cu1	130.8 (2)	C2—C7—H7	119.6
N1—O2—C8	110.3 (2)	O2—C8—C9	106.1 (3)
N1—C1—C2	125.0 (3)	O2—C8—H8A	110.5
N1—C1—H1	117.5	C9—C8—H8A	110.5
C2—C1—H1	117.5	O2—C8—H8B	110.5
C3—C2—C7	119.8 (3)	C9—C8—H8B	110.5
C3—C2—C1	122.0 (3)	H8A—C8—H8B	108.7
C7—C2—C1	118.2 (3)	C8—C9—H9A	109.5
O1—C3—C2	124.3 (3)	C8—C9—H9B	109.5
O1—C3—C4	118.4 (3)	H9A—C9—H9B	109.5
C2—C3—C4	117.3 (3)	C8—C9—H9C	109.5
C5—C4—C3	122.0 (3)	H9A—C9—H9C	109.5
C5—C4—H4	119.0	H9B—C9—H9C	109.5
			
O1^i^—Cu1—N1—C1	168.7 (3)	C7—C2—C3—O1	−178.8 (3)
O1—Cu1—N1—C1	−11.3 (3)	C1—C2—C3—O1	2.3 (5)
O1^i^—Cu1—N1—O2	4.8 (2)	C7—C2—C3—C4	0.9 (5)
O1—Cu1—N1—O2	−175.2 (2)	C1—C2—C3—C4	−178.0 (3)
N1^i^—Cu1—O1—C3	−168.8 (3)	O1—C3—C4—C5	179.6 (3)
N1—Cu1—O1—C3	11.2 (3)	C2—C3—C4—C5	−0.2 (6)
C1—N1—O2—C8	113.3 (3)	C3—C4—C5—C6	−0.2 (6)
Cu1—N1—O2—C8	−80.4 (3)	C4—C5—C6—C7	0.0 (5)
O2—N1—C1—C2	174.8 (3)	C4—C5—C6—Br1	179.3 (3)
Cu1—N1—C1—C2	9.4 (5)	C5—C6—C7—C2	0.7 (5)
N1—C1—C2—C3	−2.9 (5)	Br1—C6—C7—C2	−178.6 (2)
N1—C1—C2—C7	178.1 (3)	C3—C2—C7—C6	−1.2 (5)
Cu1—O1—C3—C2	−9.0 (5)	C1—C2—C7—C6	177.7 (3)
Cu1—O1—C3—C4	171.2 (3)	N1—O2—C8—C9	172.7 (3)

Symmetry codes: (i) −*x*+1, −*y*+1, −*z*+1.
